# Prediction of B cell and T‐helper cell epitopes candidates of bovine leukaemia virus (BLV) by in silico approach

**DOI:** 10.1002/vms3.307

**Published:** 2020-06-26

**Authors:** Negar Hooshmand, Jamal Fayazi, Saleh Tabatabaei, Nader Ghaleh Golab Behbahan

**Affiliations:** ^1^ Animal Science Department Agricultural Sciences and Natural Resources University of Khuzestan Mollasani Iran; ^2^ Razi Vaccine and Serum Research Institute Agricultural Research Education and Extention Organization (AREEO) Tehran Iran

**Keywords:** bioinformatic, BLV virus, epitope, gp60 protein, vaccine designing

## Abstract

The bovine leukaemia virus (BLV) is a retrovirus responsible for enzootic bovine leukaemia (EBL) disease, the most common cattle disease leading to high annual economic losses to the cattle breeding industry. Virus monitoring among the sheep and cattle herds is usually done by vaccination. Inactivated virus vaccines can partially protect the livestock from viral challenge. However, vaccinated animals are likely to be infected. So, there is an essential need for producing vaccine by other methods. Gp60 SU, encoded by Env gene, is the surface glycoprotein of BLV detected to be the major target for the host immunity against the virus. Different stages were performed to predict the potential B and T‐helper cell epitopes. The general framework of the method includes retrieving the amino acid sequence of gp60 SU, conducting the sequence alignment, getting the entropy plot, retrieving the previously found epitopes, predicting the hydropathy parameters, modelling the tertiary structure of the glycoprotein, minimizing the structure energy, validating the model by Ramachandran plot, predicting the linear and discontinuous epitopes by various servers and eventually choosing the consensus immunogenic regions. Ramachandran plot scrutiny has demonstrated that the modelled prediction is accurate and suitable. By surveying overlaps of various results, 4 and 2 immunogenic regions were selected as linear and conformational epitopes respectively. Amino acids 35–53, 67–97, 288–302 and 410–421 and those of numbers 37–58 and 72–100 were the regions selected as linear and conformational epitopes respectively. The tertiary structure of the final epitope was modelled as well. A comparison of the predicted epitopes structure with that of gp60 SU envelope, illustrated that the tertiary structure of these epitopes does not change after being separated from the primary complete one. The present achievements will lead to a better interpretation of the antigen–antibody interactions against gp60 in the designing process of safe and efficient vaccines.

## Graphical abstract

Bovine leukaemia virus (BLV) is a retrovirus responsible for Enzootic Bovine Leukaemia disease, an economically important disease for cattle breeding industry. B cell and T‐helper cell epitopes of BLV were predicted. The results of the current study can be used to develop novel, safe, effective and precise vaccines against BLV.

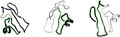

## 1 INTRODUCTION

Enzootic bovine leukaemia (EBL) is the most common neoplasia and prevalent disease of cattle, caused by a retrovirus called bovine leukaemia virus (BLV; Ghysdael et al., 1984). The EBL disease is one of the most prevalent diseases causing high economic losses to the cattle breeding industry each year (Gillet et al., 2007).

BLV is usually controlled by vaccination among sheep and cattle herds. Inactivated virus vaccines are obtained from persistently infected cell lines. The inactivation process is done through treating the virus with various chemical agents. Inactivated virus vaccines, including specific neutralizing humoral response, partially protect sheep and cattle from low dose viral challenge. However, vaccinated animals are infected with high challenge doses. Thus, it is of high necessity to develop vaccines using other methods (Rodríguez et al., 2011).

Many researches have been conducted on the different proteins of the virus. Altaner, Ban, Altanerova, and Janik (1991) illustrated the vaccination with dissimilar alive cells of the virus which lead to the productions of *Env* gene (gp60 SU surface glycoprotein) in the body protecting the cattle against bovine Leukaemia (Altaner et al., 1991). Portetelle et al. (1991) surveyed the gene of BLV surface proteins. They demonstrated that the vaccination with *Env* vaccine recombinants is capable of protecting the cattle against infection (Portetelle et al., 1991).

Experimental approaches to predict immunogenic residues are too expensive and time consuming. Several computational tools have been developed in order to predict B and T‐helper cell epitopes (Yang & Yu, 2009). One of the essential challenges in the field of in silico vaccine design is the epitopes prediction (Almagro, 2004).

According to the above‐mentioned points about the immunogenicity of gp60 SU envelope glycoprotein and its function in causing EBL disease, the present investigation aims to predict regions involved with B and T‐helper cell immune responses against gp60 SU envelope glycoprotein through in‐silico approaches. The results lead to a better interpretation of the antigen‐antibody interactions against the envelope glycoprotein, thus assisting in the design of more efficient vaccines (Haste Andersen, Nielsen, & Lund, 2006). First, linear epitopes are predicted by different servers. Second, modelling of the tertiary structure of gp60 SU is conducted. Energy minimization and validation of the structure are checked before predicting the conformational epitopes. In addition to the linear and conformational epitopes, the entropy plot, sequence alignment, previously found epitopes and hydropathy parameters are achieved as well. Finally, the consensus reliable immunogenic regions of gp60 SU envelope glycoprotein are chosen.

## 2 MATERIAL AND METHODS

### 2.1 Sequence retrieval

The complete amino acid sequence related to the reference gp60 SU one with the accession number of Q77YG2, was obtained from UniProtKB database (accessible via the link https://www.uniprot.org/help/uniprotkb). The other 895 Env protein sequences corresponding to BLV were also retrieved from the database.

Four different parts of the reference sequence were not mentioned in the server annotation. Although, 100% similar sequence with the accession number of P51519, was mentioned. The alignment of the sequences Q77YG2 (reference sequence) and P51519 was performed by BioEdit 7.9 software (Ma et al., 2019). This was done to make sure that the sequences are 100% similar. Then, according to the server annotation of P51519 sequence, four different parts of the reference sequence, (signal peptide, extracellular, helical and cytoplasmic sequences) were clarified. Also, according to UniProtKB annotations presented for the P51519 sequence, post translational modifications (PTMs) were diagnosed.

Hydropathy parameters (hydrophobicity or hydrophilicity scales) of gp60 SU glycoprotein were predicted by ProtScale tool of ExPASy server (accessible via the link https://web.expasy.org/protscale/; Gasteiger et al., 2005).

### 2.2 BLAST

BLAST was done using the NCBI server (accessible via the link https://blast.ncbi.nlm.nih.gov/Blast.cgi) in order to find regions of similarity between the biological sequences. In this way, other species that may be affected by the predicted epitopes can be found. In fact, the vaccine strain coverage can be found by BLAST. The server compares the sequence with a database of all available sequences and similar sequences with higher than a certain threshold can be obtained (Boratyn et al., 2013). This has been accomplished by the protein to protein tool (being available in the server). The none‐redundant protein sequences (nr) has been used as the default database.

### 2.3 Alignment

The protected regions of the reference sequence have been obtained by alignment with the aid of BioEdit 7.9 software. The entropy plot has been also presented by this software (Haokip et al., 2016; Ziv & Merhav, 1993).

### 2.4 Modelling, energy minimization and validation of the tertiary structure

As mentioned before, the reference sequence contains four parts. The signal peptide is not transcribed, thus having no impact on the epitope structure. The helical part of the protein is transmembrane without any direct contact with the antibodies. Also, the cytoplasmic part is located in the cell without any relation with the environment outside it. Thus, the extracellular part of the sequence, amino acids number 34–436, is the only part of the protein with the potential of being immunogenic. For this reason, only amino acids number 34–436 were used for the tertiary structure modelling (Chou, 2004). Since, the structure could not be found in PDB server or other reliable databases, the tertiary structure of gp60 SU structure was modelled by I‐TASSER (accessible via the link https://zhanglab.ccmb.med.umich.edu/I‐TASSER/). This server, working based on the multiple‐threading alignments and iterative template fragment assembly simulations, has been ranked as the best one for modelling the 3‐D protein structure in recently conducted surveys. C‐score (confidence score) presented by the server, defines the quality of the predicted models. Higher level of C‐score points out to the high confidence of the model. TM‐score and RMSD (room‐mean‐square deviation) are the standards used for measuring the structural similarity between two structures. There is a strong correlation among C‐score, TM‐score and RMSD. The lower values of RMSD and TM‐score indicate better fit and high‐resolution models (Wu, Skolnick, & Zhang, 2007).

The energy minimization was done by Swiss‐PdbViewer (Guex & Peitsch, 1997; Johansson et al., 2012). Ramachandran validation was also performed by Rampage (accessible via the link http://mordred.bioc.cam.ac.uk/~rapper/rampage.php; Lovell et al., 2003).

### 2.5 Prediction of the linear epitopes

Prediction of the linear epitopes was carried out using different servers, including ABCpred (Han et al., 2015), BcePred (Saha & Raghava, 2004), IEDB (Immune Epitope Database and Analysis Resource; Zhang et al., 2008), CBTOPE (Ansari & Raghava, 2010), SVMTriP (Yao et al., 2012) and LBtope server (Singh, Ansari, & Raghava, 2013). Also, Ellipro server (Ponomarenko et al., 2008), was employed to predict the discontinuous epitopes further to the linear ones.

The aim of ABCpred server (accessible via the link http://crdd.osdd.net/raghava/abcpred/) is to predict B cell epitopes in an antigen sequence, using artificial neural network. This server is developed based on the recurrent neural network (machine based technique) using the fixed length patterns. It also uses a threshold between +0.1 to +1.0. An increment in the threshold value, results in better specificity but worse sensitivity. Another simulation study like the present one, indicated an accuracy of 65.93% with equal sensitivity and specificity using window length of 16 at the threshold value of 0.5. So, a default value of 0.51 was used and the default windows size was considered to be 16 (Han et al., 2015; Saha & Raghava, 2006). The predicted B‐cell epitopes were ordered by their score obtained by the trained recurrent neural network (Han et al., 2015). Data with higher antigenicity are closer to our predictive results. So we selected the first 10 data as the most relevant results and used them in our analysis (Ebrahimi et al., 2019).

BcePred server (accessible via the link http://crdd.osdd.net/raghava/bcepred/) evaluates the performance of existing linear B‐cell epitope prediction methods based on the physico–chemical properties on a non‐redundant dataset. The properties used here are including the hydrophilicity (Parker, Guo, & Hodges, 1986), flexibility (Karplus & Schulz, 1985), accessibility (Emini et al., 1985), turns (Pellequer, Westhof, & Van Regenmortel, 1993), exposed surface (Janin et al., 1978) polarity (Ponnuswamy, Prabhakaran, & Manavalan, 1980) and antigenic propensity (Kolaskar & Tongaonkar, 1990). The threshold value varies within the range of −3 to +3. The increasing threshold leads to better specificity but worse sensitivity. The default thresholds for different parameters have been selected based on the best sensitivity and specificity obtained (Saha & Raghava, 2004). This default value was taken as 1.8 for the current study.

IEDB server (accessible via the link http://www.iedb.org/) is a freely available resource cataloging the experimental data on antibody and T‐cell epitopes investigated in humans, non‐human primates and other animal species in the context of infectious disease, allergy, autoimmunity and transplantation. The server also contains tools to assist in predicting and analysing the epitopes (Zhang et al., 2008). The present prediction method was Bepipred Linear Epitope Prediction (Larsen, Lund, & Nielsen, 2006). As stated before, there is a relation among the threshold and sensitivity/specificity of the prediction method. The server threshold of 0.35 was used here which results in the sensitivity and specificity of 0.49 and 0.75 respectively (Chou, 1978).

CBTOPE server (accessible via the link http://crdd.osdd.net/raghava/cbtope/) predicts the discontinuous B‐cell epitope without any homology, just using the antigen primary sequence. This server uses amino acid composition as an input feature. The prediction accuracy of the server has been reported to be 85%. During the prediction, each amino acid of the sequence gets a SVM score. The users need to set a threshold value. Amino acids with SVM scores above this threshold would be considered as epitope, otherwise non‐epitope. By default, it is −0.3 which was observed during the server development. Using the default threshold, leads to high specificity in the prediction (Ansari & Raghava, 2010). Thus, the SVM threshold used in current study was taken as −0.3.

SVMTriP server (accessible via the link http://sysbio.unl.edu/SVMTriP/) has developed a new method in which support vector machine (SVM) has been utilized by combining the tri‐peptide similarity and propensity scores (SVMTriP) in order to achieve a better prediction performance. Using sequences with 20 amino acids leads to a sensitivity (Sn) of 80.1% and precision (P) of 55.2%. The mentioned sensitivity and precision values are the ideal ones for the server (Yao et al., 2012), thus this sequence length has been applied in the current research.

LBtope server (accessible via the link http://crdd.osdd.net/raghava/lbtope/) has developed several models using various techniques (e.g. SVM and IBk) on a large dataset of epitopes and non‐epitopes (12,063 epitopes and 20,589 non‐epitopes obtained from IEDB database). During the prediction, each overlapping 20‐mers of the sequence is given a SVM score. The scores are scaled from 20% to 100%. User need to set the probability threshold (%) above which epitopes should be printed. By default, this threshold is 60%, as this value or above ones results in high specificity in the prediction (Singh et al., 2013). Therefore, this percentage was used in the current study.

### 2.6 Prediction of the conformational epitopes

Prediction of the conformational epitopes was performed by two servers, including DiscoTope 2.0 (Kringelum et al., 2012) and Ellipro (Ponomarenko et al., 2008).

Discotope 2.0 server (accessible via the link http://www.cbs.dtu.dk/services/DiscoTope/) predicts the discontinuous B cell epitopes from the protein's 3‐D structures. The method utilizes the calculation of surface accessibility (estimated in terms of the contact numbers) and a novel epitope propensity amino acid score. The final scores are calculated by combining the propensity scores of residues in spatial proximity and the contact numbers. The different thresholds for the Discotope 2.0 score can be translated into the different sensitivity/specificity values. The lower threshold amounts correspond to the higher sensitivity. The threshold value of −3.7 has been applied here as it was suggested to yield the best sensitivity/specificity combination, resulting in the sensitivity of 0.47 (Kringelum et al., 2012).

The Ellipro server (accessible via the link http://tools.iedb.org/ellipro/) predicts the linear and discontinuous antibody epitopes on the basis of the protein antigen's 3‐D structure. ElliPro accepts the protein structure in PDB format as an input. The server associates each predicted epitope with a score, defined as a PI (protrusion index) value averaged over epitope residues. The discontinuous epitopes are defined based on the PI values and clustered based on the distance R between the residue's centres of mass (in Å). The larger R value is associated with the larger discontinuous epitopes being predicted. The minimum score and maximum distance should be declared to be able to use the server calculation. The minimum score falls within the range of 0.5–1.0. Higher scores predict fewer epitopes and vice versa. The suggested minimum score of the server is 0.5 which has been applied here. Also, the maximum distance ranges from 4 to 8 Å. Longer distances lead to the prediction of discontinuous epitopes which span larger regions and vice versa. The distance of 6 Å being suggested as the best maximum distance, was applied here. Ellipro server has been also reported to predict the linear epitopes (Ponomarenko et al., 2008).

### 2.7 Previously found epitopes

As mentioned before, IEDB server is an important resource of epitopes. It has been attempted to search for epitopes relating to BLV among the epitope sequences existing in the server, in order to compare them with the present results (Zhang et al., 2008).

### 2.8 Final sequences

Having the results of linear and discontinuous predicted epitopes, entropy plot, sequence alignment, previously found epitopes and hydropathy parameters, the best sequences can be selected as the probable epitopes. The predicted epitopes were linked to each other by the appropriate linkers.

In silico predicted epitopes must be connected to each other using the empirical linkers. Gly linkers are the most frequent flexible ones (Chen, Zaro, & Shen, 2013). Hence, ‘GGG’ linker was used to link the linear epitopes to each other. Flexible linkers contain small amino acids which lead to the flexibility, movement or interaction for domains. Incorporating Ser linkers, hydrogen bonds with water molecules will be formed. Thus, not only the linker stability in the aqueous solution will be maintained, but also unfavourable interaction between the linker and protein moieties will be reduced as well (Chen et al., 2013). Therefore, ‘GGSSGG’ linker was used to link the conformational epitopes to each other.

### 2.9 Final structure

Modelling, energy minimization and validation of the predicted B‐cell epitope structure was performed by I‐TASSER, Swiss‐PdbViewer and Rampage respectively. Then, the 3‐D structure of the predicted discontinuous epitope was compared with that of the extracellular part of the reference sequence, in order to ensure that the 3‐D structural shape of the epitope (being separated from the complete protein structure) remains unchanged with respect to that when located in the complete protein structure.

## 3 RESULTS

### 3.1 Sequences

The length of the reference sequence with accession number of Q77YG2, is 515 amino acids. As mentioned earlier, the reference sequence contains four parts. Amino acid numbers corresponding to the signal peptide, extracellular, helical and cytoplasmic sequences are 1–33, 34–436, 437–457 and 458–515 respectively.

Glycosylation, lipidation and disulphide bonds were post translational modifications mentioned in UniProtKB server notifications. Glycosylation occurs on amino acids number 129, 203, 230, 251, 256, 271, 287, 351 and 398, while the lipidation location is on amino acid number 455. Also, the disulphide bonds occur on amino acids number 212–392 and 384–391.

**FIGURE 1:**
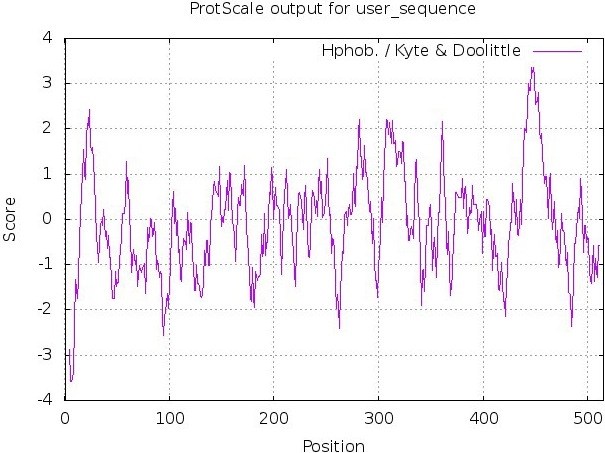
Hydropathy results of gp60 SU glycoprotein

The result of hydropathy of gp60 SU glycoprotein is demonstrated by Figure 1.

### 3.2 Blast

The list of 100 sequence accession numbers presented by BLAST tool of NCBI server is:

NP_056899.1, AFQ60883.1, AAM28597.1, AFQ00699.1, AAC18396.1, P25507.1, ABL86264.1, P25506.1, AHH24467.1, AFQ00700.1, AHH24468.1, BAU59035.1, BAU59316.1, BAU59169.1, ABL86309.1, AAK85430.3, AUV64391.1, AYM47473.1, AFQ00702.1, BAU59041.1, AAK85429.3, BAU58987.1, BAV93974.1, BAV17965.1, BAU59016.1, ABL86319.1, BAU59057.1, BAU59029.1, BAU59178.1, P25057.1, ABL86274.1, ABL86304.1, AIY70189.1, AXL95025.1, ABL86349.1, ABL86344.1, BBJ34232.1, AYM47465.1, BAV93968.1, BAV17973.1, BAX04204.1, P25505.1, AQP31221.1, P25504.1, AYM47464.1, AYM47463.1, AQP31218.1, CCJ67616.1, ABL86294.1, BAX04069.1, ABL86384.1, CCJ67622.1, AYM47453.1, BAX04240.1, AXL95013.1, AYM47472.1, AAN46089.1, ABL86289.1, AUV64402.1, AYM47454.1, AYM47457.1, BAX04105.1, AAL78062.1, ABL86364.1, AUV64394.1, BAX04060.1, AYM47459.1, AUV64405.1, AYM47467.1, AAN39879.2, ABL86369.1, AYM47461.1, AYM47451.1, BBJ34480.1, ABL86259.1, BBJ34259.1, BBJ34241.1, BBJ34340.1, BAU59289.1, BAX04222.1, ABL86339.1, AYM47452.1, BAV17981.1, BAX04267.1, BAX04258.1, BAX04096.1, BAU58993.1, AQP31216.1, BAX04276.1, BBJ34313.1, AAO21338.2, BAX04195.1, AUV64400.1, QDP17031.1, ABL86299.1, AYM47471.1, AUV64399.1, BAP46812.1, ABL86249.1, BAX04078.1

Accession numbers are sorted according to the percent identity, so that the maximum and minimum are 100 and 96.893 respectively.

### 3.3 Alignment

The alignment result is reported through the Supplementary File.

### 3.4 Modelling, energy minimization and validation of the reference tertiary structure

**FIGURE 2:**
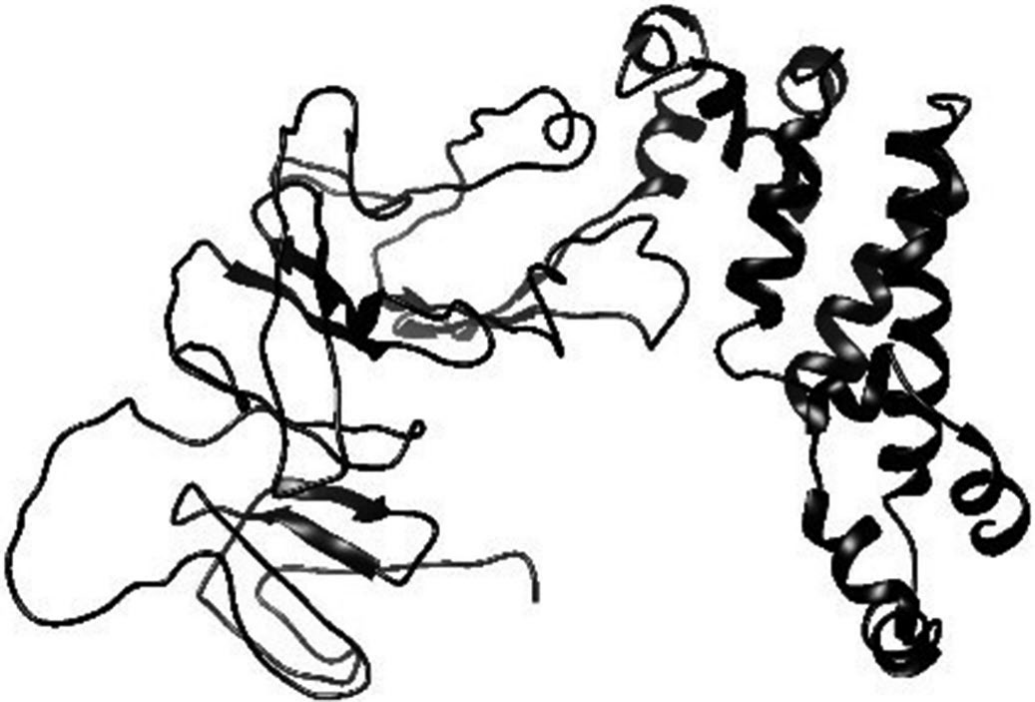
gp60 SU glycoprotein tertiary modelled structue

The Modelled structure of gp60 SU extracellular region is shown in Figure 2. The values of C‐score, estimated TM‐score and RMSD of the modelled structure were −1.50, 0.53 ± 0.15 and 10.3 ± 4.6 Å respectively. After the energy minimization, the total energy was computed as E = −18,395.275 kj/mol. The modelled structure was validated by Ramachandran plot after energy minimization. The number of residues in the favoured, allowed and outlier regions are 300 (74.8%), 67 (16.7%) and 34 (8.5%) respectively. The obtained results indicate that the structure modelling is suitable enough.

### 3.5 Entropy plot

**FIGURE 3:**
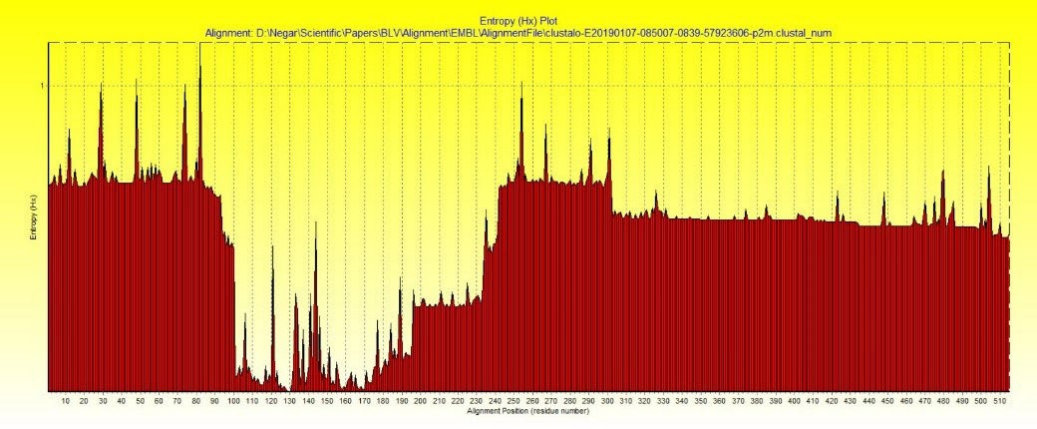
Entropy plot of gp60 SU glycoprotein

The entropy plot, presented by BioEdit 7.9 software, is demonstrated by Figure 3.

### 3.6 Prediction of the linear epitopes

The linear epitope prediction results of the above‐mentioned servers are listed in Table 1. As mentioned before, Ellipro server has also presented linear epitope prediction. The conformational epitope prediction results of Ellipro server are presented in Table 2.

**TABLE 1:** Linear epitope prediction result of gp60 SU glycoprotein

Server	Residues
Ellipro	1–20, 334–427, 72–98, 137–148, 38–69, 158–184, 486–502, 268–277, 201–212
CBtope	13–14, 35, 39–51, 62–73, 75–90, 97–124, 131, 136–138, 172–173, 179, 211–213, 214–226, 228–230, 235–237, 252–253, 256–260, 266, 268–270, 294–296, 300–302, 304, 322–323, 361, 363–371, 373, 384, 387–392, 429, 475–479, 481–483, 488, 497–498, 503–515
LBtope	30–37, 44–48, 95–97, 192–196, 203–211, 213–224, 227, 231–232, 261, 265–269, 285–292, 294, 296–302, 327–328, 330–331, 333, 349–354, 366–376, 404–405, 410, 412–427, 429–430
SVMTrip	67–86, 109–128, 216–235, 251–270, 278–297
IEDB	1–12, 45–52, 67–77, 88–99, 110–117, 121–122, 124–137, 175–193, 207–208, 215–224, 232–242, 259–264, 291–302, 409–410, 412–421, 472–490, 492, 494–498, 500–509
ABCpred	2–11, 6–15, 18–27, 27–36, 33–42, 40–49, 46–55, 52–61, 59–68, 70–79, 74–83, 81–90, 85–94, 89–98, 94–103, 99–108, 109–118, 117–126, 113–122, 134–143, 142–151, 146–155, 153–162, 157–166, 161–170, 169–178, 173–182, 180–189, 186–195, 192–201, 204–213, 214–223, 218–227, 228–237, 234–243, 243–252, 248–257, 253–262, 257–266, 261–270, 270–279, 283–292, 292–301, 297–306, 302–311, 306–315, 312–321, 316–325
BCEpred (Hydro)	5–7, 9, 50–51, 67, 131–134, 180, 414, 483–486,
BCEpred (Flexi)	1–8, 93–96, 231, 234, 297–298, 340, 365, 411, 480–481, 502
BCEpred (Access)	3–13, 32, 45–53, 67, 75–78, 87,89–91, 93–98, 100, 110, 131–132, 177–184, 264–265, 295, 297–301, 331, 341–345, 353–354, 367–370, 410–411, 414–416, 469, 472, 481–486, 502–510
BCEpred (Turns)	67–68, 123, 263
BCEpred (Surface)	1–11, 96, 178, 180, 298, 342–343, 483–485
BCEpred (Polar)	2–11, 96–101, 123, 150–151, 264–265, 298–301, 342–343, 434–436, 466
BCEpred (AntiPro)	17, 20–22, 30, 37–38, 109, 111, 113–115, 141–152, 155, 170–171, 174, 192, 194–195, 227, 269–271, 281–286, 288–289, 335–339, 357, 377, 381–384, 390–394, 411–412, 437–442, 457–459, 461–462, 465–467, 500, 511

**TABLE 2:** Conformational epitope prediction result of gp60 SU glycoprotein

Server	Residues
Ellipro	37–58, 72–76, 78–88, 91–98, 100–107, 124–129, 132–133, 135–142, 164–166, 173–196, 201, 203–213, 231–238, 273–277, 291–298, 300, 304–305, 315–316, 318–332, 350, 358, 361–378, 380–381, 384–385, 388–389, 391–421, 423–424, 426–436
Discotope 2.0	34, 40, 42–56, 73–75, 77, 79, 81–104, 106, 132, 134, 176–180, 183, 398–400, 403, 406–407, 410–411, 414, 418

### 3.7 Prediction of the conformational epitopes

The raw results associated with the conformational epitope prediction obtained via the two servers of Ellipro and Discotope 2.0, are presented in Table 2.

### 3.8 Previously found epitopes

The list of previously found epitopes retrieved from IEDB server is: 21–28 (30,853), 24–32 (79,873), 39–48 (6,834), 45–59 (489,850), 53–67 (489,463), 57–67 (76,471), 57–71 (489,594), 59–69 (48,878), 60–79 (98,281), 61–70 (98,345), 61–75 (489,736), 61–78 (49,009), 61–80 (98,346), 64–73 (55,707), 68–87 (18,750), 71–90 (15,315), 72–81 (5,994), 74–83 (67,318), 74–93 (98,184), 77–91 (489,763), 78–92 (13,743), 85–99 (489,572), 89–103 (489,601), 91–110 (489,852), 93–107 (489,710), 97–111 (489,760), 98–117 (60,362), 101–115 (489,547), 115–124 (50,183), 117–136 (68,617), 119–132 (6,385), 121–140 (20,262), 131–140 (98,087), 131–150 (98,088), 134–153 (97,952), 142–161 (31,383), 144–155 (47,160), 144–157 (47,161), 144–163 (98,117), 149–163 (489,646), 153–167 (489,575), 157–171 (489,811), 165–179 (489,854), 168–180 (37,627), 169–183 (489,599), 169–188 (38,323), 177–191 (489,690), 177–192 (47,063), 188–200 (79,953), 189–199 (227,838), 189–203 (489,768), 195–205 (72,113), 197–211 (489,797), 213–238 (1,864), 237–251 (489,557), 245–259 (489,474), 251–270 (45,990), 253–267 (489,789), 256–268 (61,995), 257–271 (489,823), 260–268 (56,937), 269–283 (489,640), 273–287 (489,782), 277–291 (489,468), 282–293 (47,985), 282–301 (47,986), 285–299 (489,662), 342–361 (489,509), 353–372 (489,807), 372–391 (489,513) and 452–471 (489,629). The parenthesized codes after residue numbers are epitope IDs presented and assigned to each epitope by IEDB server.

### 3.9 Final sequences

Gathering all information together, four protein sequences were predicted as linear epitopes, including amino acids number 35–53, 67– 97, 288–302 and 410– 421. The predicted linear epitopes, linked to each other by GGG linker, resulted in the following sequence:

CSLSLGNQQWMTTYNQEA**GGG**NQSPFCPRSPRYTLDFVNGYPKIYWPPPQGR**GGG**LSTVSSAPPTRVRRS**GGG**LSQRVSTDWQWP.

Also, the predicted conformational epitopes which are amino acids number 37–58 and 72–100, are linked to each other by GGSSGG linker and lead to the following sequence:

SLSLGNQQWMTTYNQEAKFSIS**GGSSGG**CPRSPRYTLDFVNGYPKIYWPPPQGRRRF.

Amino acids of the predicted conformational epitope regions were also predicted as the linear epitopes. Also, they were checked to be on the surface of the gp60 SU tertiary structure in order to be accessible by the antibodies.

Epitopes predicted in the current study were compared with the results of previously found epitopes retrieved from IEDB server. Comparison of the linear and conformational epitopes are given by Tables 3 and 4 respectively. The epitope IDs are reported through parentheses. All retrieved epitopes from IEDB belonging to BLV, are encoded from Env gene and related to gp60 SU glycoprotein. The first three predicted linear and both predicted discontinuous epitopes have been also reported by the previous experimental studies. It means that the current in silico prediction has been sufficiently accurate. The fourth linear epitope, amino acids number 410–421, has not been previously predicted by the experimental studies. Thus, the epitope can be considered as a novel predicted one which can be surveyed more by future experiments.

**TABLE 3:** Comparison of linear predicted epitopes with previously found epitopes retrieved from IEDB

Predicted Epitope	IEDB Epitopes
35–53	39–48 (6,834), 45–59 (489,850)
67–97	53–67 (489,463), 57–67 (76,471), 57–71 (489,594), 59–69 (48,878), 60–79 (98,281), 61–70 (98,345), 61–75 (489,736), 61–78 (49,009), 61–80 (98,346), 64–73 (55,707), 68–87 (18,750), 71–90 (15,315), 72–81 (5,994), 74–83 (67,318), 74–93 (98,184), 77–91 (489,763), 78–92 (13,743), 85–99 (489,572), 89–103 (489,601), 91–110 (489,852), 93–107 (489,710), 97–111 (489,760)
288–302	277–291 (489,468), 282–293 (47,985), 282–301 (47,986), 285–299 (489,662)
410–421	No Epitopes were found

**TABLE 4:** Comparison of conformational predicted epitopes with previously found epitopes retrieved from IEDB

Predicted Epitope	IEDB Epitopes
37–58	39–48 (6,834), 45–59 (489,850), 53–67 (489,463), 57–67 (76,471), 57–71 (489,594)
72–100	61–75 (489,736), 61–78 (49,009), 61–80 (98,346), 64–73 (55,707), 68–87 (18,750), 71–90 (15,315), 72–81 (5,994), 74–83 (67,318), 74–93 (98,184), 77–91 (489,763), 78–92 (13,743), 85–99 (489,572), 89–103 (489,601), 91–110 (489,852), 93–107 (489,710), 97–111 (489,760), 98–117 (60,362)

### 3.10 Modelling, energy minimization and validation of the conformational epitope structure

**FIGURE 4:**
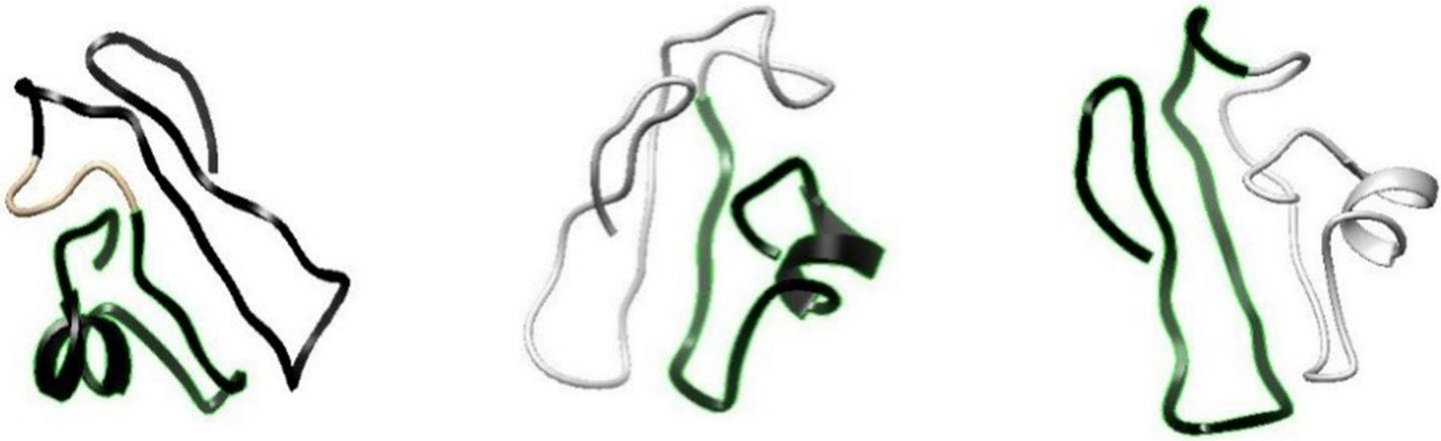
Tertiary structure of predicted B cell epitope

The modelling, energy minimization and validation of the conformational epitope structure was performed using the above‐mentioned software and servers. The estimated values of C‐score, TM‐score and RMSD are −2.95, 0.38 ± 0.13 and 9.1 ± 4.6 Å respectively. The total energy has been computed as E = −2,466.034 kj/mol. The number of residues in the favoured, allowed and outlier regions are 32 (58.2%), 19 (34.5%) and 4 (7.3%) respectively. The surveys demonstrate that the model results are of acceptable accuracy. The final model is exhibited in Figure 4. In this figure, both predicted epitopes, epitope containing amino acids number 37–58 and epitope containing amino acids number 72–100, are illustrated from left to right respectively.

The 3‐D structures of both predicted discontinuous epitopes have been compared with that of the extracellular part of the reference sequence. It has been indicated that the structures of both conformational epitopes are the same with their structure in the whole extracellular part of gp60 SU glycoprotein. Thus, the 3‐D structural shape of both epitopes remains unchanged after being separated from the primary complete structure.

## 4 DISCUSSION

As mentioned in above, BLV contains different proteins and glycoproteins. Gp60 SU glycoprotein is detected to be the main infectious agent. Therefore, the immunization with gp60 SU, can lead to the host immunity against BLV (Portetelle et al., 1991). In the current study, several computational methods have been used to predict the reliable B and T‐helper cell epitopes.

Epitopes are able to stimulate immune response. Thus, epitope identification, either by experimental or computational methods, is essential for purposes such as vaccine design, laboratory kit design, etc. (Irving, Pan, & Scott, 2001).

X‐ray crystallography, nuclear magnetic resonance (NMR) and monoclonal antibodies are introduced as the most reliable methods for the epitope identification. Since, these experimental methods are time‐consuming and costly (Yang & Yu, 2009), computational methods are considered to be the most effective approaches in order to conduct the epitope prediction ( Resende et al., 2012).

Given the results associated with the linear and discontinuous predicted epitopes, entropy plot, sequence alignment, previously found epitopes and hydropathy parameters, the best sequences have been selected as the probable epitopes and presented in Tables 1 and 2 as well. Amino acids of the predicted conformational epitope regions were also predicted as the linear one. Moreover their locations on the 3‐D structure of the glycoprotein were examined and two regions were eventually chosen as the conformational epitopes. As would be observed, both peptides were on the surface of the gp60 SU tertiary structure, indicating that the epitopes are accessible by the antibodies.

The tertiary structures of both discontinuous predicted epitopes have been compared with that of the extracellular part of the reference sequence. It has been indicated that the structures of both conformational epitopes are the same with their structure in the whole extracellular part of gp60 SU glycoprotein. Thus, the tertiary structural shape of both epitopes remains unchanged through their elicitation from the primary complete structure.

The present predicted epitopes have been compared with those via other researches retrieved from IEDB server. All retrieved epitopes from IEDB which belong to BLV, are encoded from Env gene and related to gp60 SU glycoprotein. Most epitopes related to the previous studies and compared with the present results, have been obtained by the experimental methods.

The first three predicted linear epitopes (amino acids number 35–53, 67–97 and 288–302) and both predicted discontinuous ones (amino acids number 37–58 and 72–100) were the same as those reported by the experimental studies. This further indicates the accuracy of the present in silico prediction. The predicted discontinuous epitope with amino acids number 72–100, overlaps with the epitope sequence obtained by Callebaut et al. (1991). Also, the predicted discontinuous epitopes with amino acids 37–58 and the linear ones with amino acids 35–53, are consistent with the epitope sequences reported by Portetelle et al. (1989) and Bai et al. (2015). The predicted linear epitope with amino acids 97–67, overlaps with the epitope sequence achieved by Gatei, Good, Daniel, and Lavin (1993) and Callebaut et al. (1993). Further to these, the predicted linear epitope with amino acids 288–308, overlaps with the epitope sequence obtained by Callebaut et al. (1993).

However, the fourth linear epitope with amino acids 410–421, has not been previously predicted by the experimental studies. Thus, this newly predicted epitope can be surveyed more by future experiments.

The present study has led to information about gp60 SU glycoprotein immunogenic regions which may be helpful for conducting future laboratory experiments. The present findings can be used to develop novel, safer, more effective as well as precise vaccines against BLV.

## CONFLICT OF INTEREST

The authors declare that they have no conflict of interest.

## AUTHOR CONTRIBUTION

**Negar Hooshmand**: Formal analysis; Investigation; Writing‐original draft. **Jamal Fayazi**: Conceptualization; Formal analysis; Investigation; Supervision; Writing‐review & editing. **Saleh Tabatabaei**: Data curation; Validation; Writing‐review & editing. **Nader Ghaleh Golab Behbahan**: Data curation; Methodology; Validation; Writing‐review & editing.

### ETHICS

We hereby declare all ethical standards have been respected in preparation of the submitted article.

## Supporting information

Supplementary Material

Supplementary Material
